# Global Guided Cross-Modal Cross-Scale Network for RGB-D Salient Object Detection

**DOI:** 10.3390/s23167221

**Published:** 2023-08-17

**Authors:** Shuaihui Wang, Fengyi Jiang, Boqian Xu

**Affiliations:** Changchun Institute of Optics, Fine Mechanics and Physics, Chinese Academy of Sciences, Changchun 130033, China; wangshuaihui@ciomp.ac.cn (S.W.); jiangfengyi@ciomp.ac.cn (F.J.)

**Keywords:** RGB-D salient object detection, global guidance, cross-modal cross-scale fusion

## Abstract

RGB-D saliency detection aims to accurately localize salient regions using the complementary information of a depth map. Global contexts carried by the deep layer are key to salient objection detection, but they are diluted when transferred to shallower layers. Besides, depth maps may contain misleading information due to the depth sensors. To tackle these issues, in this paper, we propose a new cross-modal cross-scale network for RGB-D salient object detection, where the global context information provides global guidance to boost performance in complex scenarios. First, we introduce a global guided cross-modal and cross-scale module named G^2^CMCSM to realize global guided cross-modal cross-scale fusion. Then, we employ feature refinement modules for progressive refinement in a coarse-to-fine manner. In addition, we adopt a hybrid loss function to supervise the training of G^2^CMCSNet over different scales. With all these modules working together, G^2^CMCSNet effectively enhances both salient object details and salient object localization. Extensive experiments on challenging benchmark datasets demonstrate that our G^2^CMCSNet outperforms existing state-of-the-art methods.

## 1. Introduction

The goal of salient object detection (SOD) is to identify the most distinctive object in a given image or video. As an important pre-processing method, SOD is widely used in various computer vision tasks, including image understanding [1], video detection and segmentation [2], semantic segmentation [3], object tracking [4], etc.

With the development of deep learning, numerous RGB SOD models have been proposed and have achieved significant success [5,6,7,8,9]. However, when dealing with complicated scenes with low texture contrast or cluttered backgrounds, the performance of RGB SOD models deteriorates. RGB-D SOD models, which extract objects from paired RGB images and depth maps, have attracted growing interest due to their complementary information and accessibility.

Although many new CNN-based SOD approaches [10,11,12,13,14,15,16,17,18] have been proposed for RGB-D and have achieved better performance than before, there are some issues affecting the performance of existing RGB-D SOD. First, although providing complementary information, depth maps occasionally contain misleading information due to the limitations of the depth sensors [12,13,19], which deteriorate the performance of RGB-D saliency models. Second, the empirical receptive field of CNN is much smaller than the theoretical one, especially on high-level layers, and semantic information carried by deep layers may be gradually diluted when transmitted to shallower layers [8,9,17]. Lack of global context increases the chances of failure in object localization. Third, previous methods usually enhance RGB features by corresponding level depth features rather than cross-scale depth features [15,16]. Zhao [17] found that it is effective to reduce errors due to the similar appearance of objects when utilizing global scene clues. Liu [8] proposed a receptive field block (RFB) to capture global context information. Liu [9] utilizes the revised Pyramid Pooling Module (PPM) as global guidance to guide RGB-D SOD. Li [14] found that cross-scale fusion between two adjacent blocks can effectively capture feature continuity and activate cross-modal cross-scale interactions. Inspired by the above findings, our work exploits a global guided cross-modal cross-scale network to alleviate the influence of low-quality depth maps for the RGB-D SOD task, which employs PPM as global guidance to enhance global context information.

In this paper, we propose a new Global Guided Cross-Modal Cross-Scale Network (G^2^CMCSNet) that significantly improves the performance of RGB-D SOD. We propose a Cross-Modal Cross-Scale Module to fuse features and embed PPM blocks into CMCSM to enrich global context information. The proposed G^2^CMCSM preserves the global contextual information of the object while maintaining its details.

Our main contributions are summarized as follows:

- We exploit cross-modal cross-scale feature fusion under the guidance of global context information to suppress distractors in lower layers. This strategy is based on the observation that high-level features contain global context information, which is helpful to eliminate distractors in lower layers.
- To fully capture the complementary information in the depth map and effectively fuse RGB features and depth features, we introduce a depth enhancement module (DEM), which utilizes the complementarity between RGB features and depth features, and an RGB enhancement module (REM) which utilizes the information of RGB features to improve the details of salient object detection.
- We propose a Global Guided Cross-Modal Cross-Scale Network (G^2^CMCSNet) to detect RGB-D salient objects, which exceed 12 SOTAs on five public datasets. Different from other models, our model not only considers feature continuity but also exploits the intrinsic structure of the RGB features and the global context information of high-level features. The performance of the proposed method is evaluated on five popular public datasets under four evaluation metrics, and compared with 12 state-of-the-art RGB-D SOD methods.

The remainder of this paper is structured as follows: We first describe the present status of salient object detection and RGB-D salient object detection in Section 2. The overall architecture, components, and loss function of the network are outlined in Section 3. Additionally, Section 4 provides the outcomes of our experiments. Finally, Section 5 presents our conclusions.

## 2. Related Work

Deep Learning-Based RGB Salient Object Detection. Owing to the development of deep learning, a great number of deep learning-based RGB salient object detection has been proposed in recent years. Compared with traditional methods, Deep Learning-Based methods have become the most mainstream due to their excellent extraction accuracy. For example, Wang et al. [18] developed a recurrent fully convolutional network for salient object detection that incorporates saliency prior knowledge for more accurate prediction. Liu et al. [20] proposed a novel end-to-end deep hierarchical saliency network that produces coarse saliency maps first and refines them recurrently and hierarchically with local context information. To compensate for the diluted global semantic information, Liu et al. [9] introduced a global guidance module and a feature aggregation module to the U-shape network. Chen et al. [21] applied side-output residual learning for refinement in a top-down manner under the guidance of a reverse attention block, which led to significant performance improvement. Wang et al. [22] presented a salient object detection method that integrates both top-down and bottom-up saliency inference iteratively and cooperatively, which encourages saliency information to effectively flow in a bottom-up, top-down, and intra-layer manner. Wu et al. [23] proposed a novel cascaded partial decoder framework for fast and accurate salient object detection that discards higher-resolution features of shallower layers for acceleration and integrates features of deeper layers for a precise saliency map. To detect objects in cluttered scenes, Zhang et al. [24] utilized image captioning to boost the semantic feature learning for salient object detection, which encodes the embedding of a generated caption to capture the semantic information of major objects and incorporates the captioning embedding with local-global visual contexts for predicting the saliency map.

The works mentioned above perform better than traditional approaches, but they fall short when it comes to complex scenes. To address this issue, deep learning-based RGB-D salient object detection is proposed.

Deep Learning-Based RGB-D Salient Object Detection. Numerous CNN-based RGB-D SOD methods [5,6,7,8,9,11,13,15,16,18,25,26,27,28,29,30,31,32,33,34,35,36,37] have been proposed with the development of depth sensors. Recent RGB-D SOD models mainly focus on CNN architectures and cross-modal fusion strategies to improve the performance of salient object detection. Fan et al. [13] proposed a multi-level, multi-modality learning framework that splits the multi-level features into teacher and student features and utilizes depth-enhanced modules to excavate informative parts of depth cues from the channel and spatial views. Li et al. [14] introduced three RGB-depth interaction modules to enhance low-, middle-, and high-level cross-modal information fusion. Zhang et al. [26] proposed a multi-stage cascaded learning-based RGB-D saliency detection framework that explicitly models complementary information between RGB images and depth data by minimizing the mutual information between modalities. Wang et al. [34] introduced correlation fusion to fuse RGB and depth correlations and long-range cross-modality correlations and local depth correlations to predict salient maps. Bi et al. [35] introduced a cross-modal hierarchical interaction network that boosts salient object detection by excavating the cross-modal feature interaction and progressively multi-level feature fusion. Zhang et al. [37] utilized the dynamic enhanced module to dynamically enhance the intra-modality features and the scene-aware dynamic fusion module to realize dynamic feature selection between two modalities.

Apart from the fusion architectures, the quality of the depth map also affects the performance of salient object detection. Ji et al. [24] designed a depth calibration strategy to correct the potential noise from unreliable raw depth. Sun et al. [29] introduce a depth-sensitive RGB feature modeling scheme using the depth-wise geometric prior to reducing background distraction. Zhang et al. [30] designed a convergence structure that effectively selects the most valuable supplementary information from RGB and depth modalities to obtain a more discriminative cross-modality saliency prediction feature. Wu et al. [31] proposed a new fusion architecture for RGB-D saliency detection, which improves robustness against inaccurate and misaligned depth inputs.

## 3. Methodologies

In this section, we present the overall architecture and motivation of the proposed Global Guided Cross-Modal Cross-Scale Network (G^2^CMCSNet) in Section 3.1. In Section 3.2, we introduce its main components in detail, including G^2^CMCSM and feature refinement modules.

### 3.1. The Overall Architecture and Motivation

**Figure 1:**
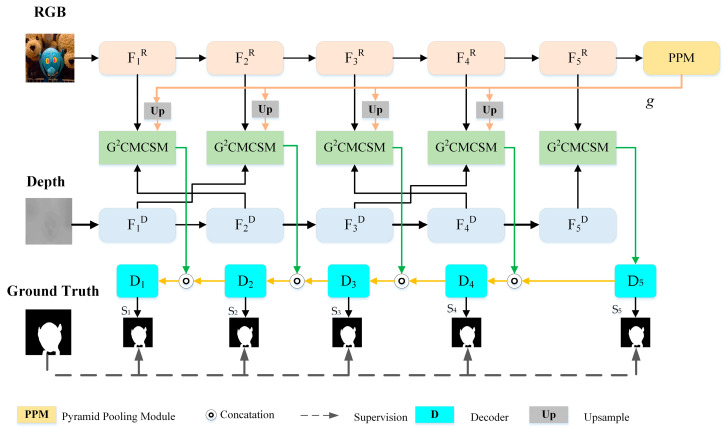
The overall architecture of the proposed network. First, the feature encoding network extracts features from the RGB image and depth map. Then, these features and outputs of the Pyramid Pooling Module (PPM) are fed to the Global Guided Cross-Modal Cross-Scale Module (G^2^CMCSM) for feature fusion. Finally, the outputs of G^2^CMCSM modules are transferred to cascaded decoder blocks for progressive refinement.

The proposed G^2^CMCSNet follows the encoder-decoder structure. As illustrated in Figure 1, G^2^CMCSNet consists of a feature encoder, global guided cross-modal cross-scale interaction, and global guided feature refinement.

Feature Encoder. We employ ResNet-50 as the backbone to extract RGB and depth features, which have been pre-trained on the ImageNet dataset. As shown in Figure 1, the RGB image and depth map are encoded separately through the two-stream ResNet-50. These encoded blocks of the RGB image and depth map are denoted by $f_{n}^{D}$ and $f_{n}^{R}$(*n* ∈ {1, 2, 3, 4, 5}) is the block index), respectively. The input resolutions of RGB image and depth map are set to 256 × 256 × 3 and 256 × 256 × 1, respectively.

Global Guided Cross-Modal Cross-Scale Module. It has been proven in [14] that adjacent feature fusion of low- and mid-level features can effectively capture feature continuity and promote cross-modal and cross-scale fusion. Meanwhile, high-level semantic features help discover the specific locations of salient objects. Based on the above knowledge, we propose global guided cross-modal modules to capture the exact positions of salient objects while sharpening their details at the same time.

It has been proven that features extracted from the deep layer contain rich semantic and textural information [17]. Meanwhile, depth maps occasionally contain misleading information, which deteriorates the performance of RGB-D saliency models. The Pyramid Pooling Module (PPM) provides global contextual information. Therefore, PPM is introduced as global guidance and embedded in the final RGB feature layer, and the output is employed to enhance global context information.

Cascaded Decoder. As we know, shallow layers contain low-level structure cues, while deep layers contain global semantic information. Therefore, we adopt a cascaded decoder to refine our saliency maps progressively. As shown in Figure 1, the cascaded decoder module consists of five decoder blocks, which receive outputs from G^2^CMCS for refinement. The initial saliency map S_5_ with high-level features is progressively refined by low-level features, which contain abundant detailed information.

### 3.2. Global Guided Cross-Modal Cross-Scale Module

**Figure 2:**
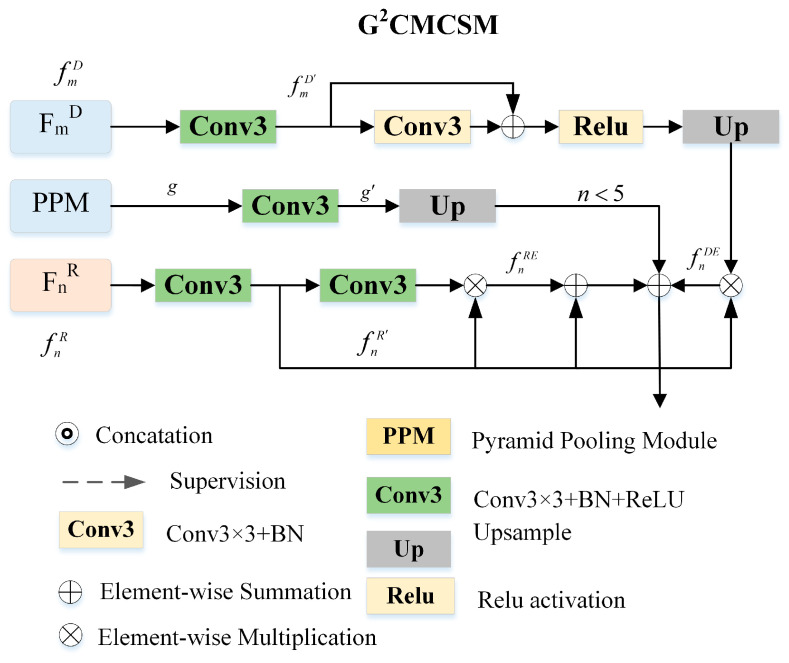
Architecture of the global guided cross-modal cross-scale module (G^2^CMCSM). The module receives two input features from RGB and depth branches to exchange the multimodal features and produce the cross-modal cross-scale feature.

The global guided cross-modal cross-scale module is the key component of G^2^CMCSNet, which integrates the cross-modal and cross-scale information under the guidance of global context information. The details of G^2^CMCS are illustrated in Figure 2.

First, depth maps may contain misleading information due to their low-quality. Second, global information is diluted when transferred to shallower levels. Based on the above facts, our fusion strategy employs the details of RGB features and locations of depth features. Therefore, G^2^CMCS includes three parts, Depth Enhancement Module (DEM), RGB Enhancement Module (REM), and Global Guidance (GG).

Depth Enhancement Module (DEM). To reduce the noise of low-quality depth maps, we introduce a depth enhancement module (DEM) to improve the compatibility of multi-modal features. Different from other works, we divide the first four CNN layers into low-level parts and the last CNN block into high-level parts. The low-level RGB features of low-level are enhanced by adjacent level depth features, while the high-level RGB features are enhanced by their corresponding level depth features.

Specifically, taking $f_{n}^{R}$ and $f_{m}^{D}$ as an example, the relation between m and n can be expressed as follows:

(1) $$m=\left\{\begin{array}{l} n+1,n=1,3\\ n-1,n=2,4\\ n,n=5 \end{array}\right.$$

For channel reduction, the two features are fed into a 3 × 3 convolutional layer with BatchNorm and Relu activation, as shown in Figure 2. Thus, we can obtain the normalized feature maps $f_{n}^{R^{\prime }}$ and $f_{m}^{D^{\prime }}$. The depth branch $h_{m}^{D}$ contains two branches to enlarge the receptive field and a residual connection to retain the original information.

(2) $$h_{m}^{D}=Relu\left(f_{m}^{D^{\prime }}+Conv_{3\times 3}(f_{m}^{D^{\prime }})\right),n\in [1,5]$$

where $Conv_{3\times 3}\left(\cdot \right)$ denotes a 3×3 convolution with BatchNorm, $Relu\left(\cdot \right)$ is the Rule activation function. Then, we have the DEM representations as follows:

(3) $$f_{n}{}^{DE}=f_{n}^{R^{\prime }}⊗h_{m}^{D},n,m\in [1,5]$$

where ⊗ denotes element-wise multiplication.

RGB Enhancement Module (REM). RGB features contain information such as color and texture, which helps sharpen the details of SOD. Therefore, we propose an RGB enhancement module (REM) to enhance the details of SOD.

Specifically, $f_{n}^{R^{\prime }}$ is fed into a 3 *×* 3 convolutional layer with BatchNorm and Relu activation and is enhanced by itself. The REM can be representations are defined as follows:

(4) $$f_{n}{}^{RE}=f_{n}^{R^{\prime }}⊗BConv_{3\times 3}(f_{n}^{R^{\prime }}),n\in [1,5]$$

where $BConv_{3\times 3}\left(\cdot \right)$ denotes a 3×3 convolution with BatchNorm and Relu activation.

Global Guidance (GG). PPM is employed as a global guidance module for its global context information. Global context information carried by the highest level is skipped and connected to the low-level part to remedy diluted global information. PPM is placed on top of the backbone of the RGB feature stream to capture global context information. Meanwhile, skip connection is used to remedy diluted global context information. Considering that high-level RGB features contain global context information, PPM is connected to a low-level part of the network.

To preserve the original information carried by the RGB feature, residual connections are adapted to combine the enhanced features with the original RGB features. We apply element-wise summation to fuse features, and the cross-modal cross-scale enhanced feature $f_{n}^{RD}$ representations are defined as follows:

(5) $$f_{n}{}^{RD}=\left\{\begin{array}{l} =f_{n}^{R^{\prime }}+f_{n}^{DE}+f_{n}^{RE}+Up(g^{\prime }),n\in [1,4]\\ =f_{n}^{R^{\prime }}+f_{n}^{DE}+f_{n}^{RE},n=5 \end{array}\right.$$

where + denotes element-wise summation, $Up\left(\cdot \right)$ denotes upsample operation. A 3×3 convolutional layer is followed to obtain smooth features.

Our G^2^CMCSM can capture the exact positions of salient objects and sharpen their details at the same time with skip connections.

### 3.3. Cascaded Decoder

To effectively leverage the features, the network is decoded with a cascaded refinement mechanism. This mechanism first produces an initial saliency map with high-level features and then improves the details of the initial saliency map $S_{5}$ with low-level features that contain abundant detailed information. Using this mechanism, our model can iteratively refine the details in the low-level features. Corresponding to the five-level cross-modal cross-scale fusion, the cascaded decoder is a five-level module that consists of five decoders, as shown in Figure 1. Each decoder contains 3×3 convolution layers and upsamples layers. Finally, we obtained the final prediction map $S_{1}$, which can be donated as follows:

(6) $$S_{n}=\left\{\begin{array}{l} D\left(Up\left(S_{n+1}\right),S_{n}\right),n\in [1,4]\\ D\left(f_{5}^{RD}\right),n=5 \end{array}\right.$$

where $D\left(\cdot \right)$ denotes decoder operation, $S_{n}$ denotes prediction map, $Up\left(\cdot \right)$ denotes upsample operation, and $S_{1}$ denotes the final output.

### 3.4. Loss Function

We supervise multi-level outputs to take advantage of fusion information. Loss is composed of the Binary Cross-Entropy (BCE) loss $L^{BCE}$ and the Intersection-Over-Union (IOU) loss $L^{IOU}$ [38]. As shown in Figure 1, each $S_{n}$ is supervised by the GT during the BCE loss and IOU loss phases. The total loss function *L* can be formulated as follows:

(7) $$L=\sum_{n=1}^{5}(L_{n}^{BCE}+L_{n}^{IOU})$$

## 4. Experiments

### 4.1. Datasets and Evaluation Metrics

Datasets. To verify the effectiveness of our method, we evaluated the proposed method on five widely used benchmark datasets, including STEREO [39], NJU2K [38], NLPR [40], SSD [41], and SIP [42]. To make a fair comparison, we followed the same training settings as existing works [12], which consist of 1485 samples from NJUD and 700 samples from NLPR.

Evaluation Metrics. We evaluate the performance of our method and other compared methods using four widely used evaluation metrics, including maximum F-measure ($F_{\beta }$, $\beta^{2}=0.3$) [43], mean absolute error (MAE, M) [44], S-measure ($S_{\lambda }$, λ=0.5) [45], and maximum E-measure ($E_{\xi }$) [46]. To make a fair comparison, we use the tools provided by [16] to evaluate each SOTA method.

### 4.2. Implementation Details

Our model is implemented on the basis of PyTorch with one NVIDIA A4000 GPU. The backbone network (ResNet-50) is used, which has been pre-trained on ImageNet. Due to the different channels of RGB and depth images, the input channel of the depth encoder is set to 1. The proposed model is optimized by the Adam algorithm; the initial learning rate is set to 10^−4^ and is divided by 10 every 60 epochs. All training and testing images are resized to 256 × 256. The training images are augmented using various strategies, including random flipping, rotating, and border clipping. The batch size is set to 10, and the model is trained for 120 epochs.

### 4.3. Comparison with State-of-the-Art

Quantitative Comparison. We compare the proposed network with 14 state-of-the-art (SOTA) CNN-based methods, which are DMRA [47], CMW [14], PGAR [12], HAINet [15], JLDCF [33], DCF [27], DSA2F [29], DCMF [34], HINet [35], CFIDNet [36], SPSNet [28], and C2DFNet [37]. The saliency maps of all compared methods are provided by the authors or obtained by running their released codes. Table 1 shows the quantitative comparison in terms of four evaluation metrics on five datasets. It can be seen that G^2^CMCSNet significantly outperforms the competing methods across all the datasets in most metrics. Especially, G^2^CMCSNet outperforms all other methods by a dramatic margin on the LFSD and SIP datasets, which are considered more challenging datasets. Moreover, G^2^CMCSNet consistently surpasses all other state-of-the-art methods in five datasets in terms of the overall performance metric. Overall, our proposed G^2^CMCSNet obtains promising performance in locating salient object(s) in a given scene.

**Figure 3:**
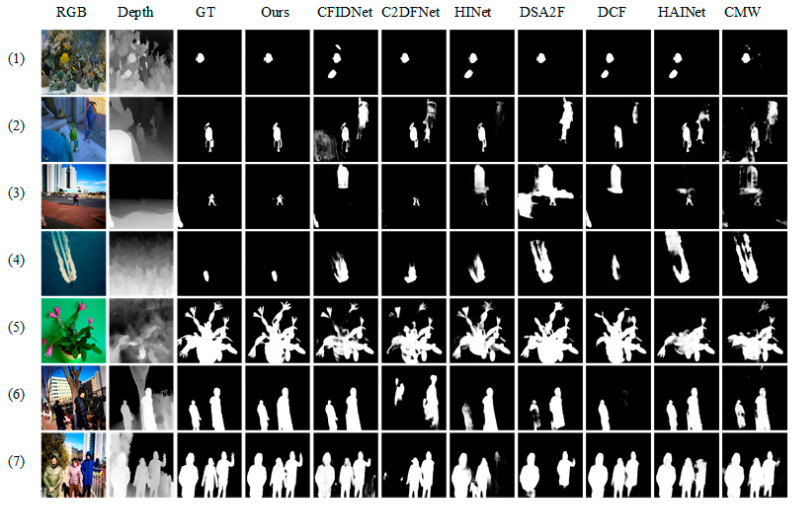
Visual comparison of our method and seven SOTAS (including CMW, HAINet, DCF, DSA2F, HINet, C2DFNet, and CFIDNet).

Qualitative Comparison. Figure 3 shows qualitative comparisons with seven representative methods on various challenging scenarios for SOD. The first row represents a scene with low contrast. Our method and DSA2F accurately capture the salient object while others failed. In the second and third rows, our method successfully locates small objects with complex backgrounds. In the fourth row, we represent a scene with a low-quality depth map. It can be seen that our method captures the salient object by eliminating the misleading information of low-quality depth maps. The sixth and seventh rows show scenes with multiple objects and our method produces the most reliable results. Thanks to the proposed G^2^CMCSM and refinement strategy, our method can accurately highlight salient objects regardless of complicated scenes and low-quality depth maps.

### 4.4. Ablation Study

To verify the relative contribution of different components of our model, we carry out ablation studies by removing or replacing them from our full model.

**Figure 4:**
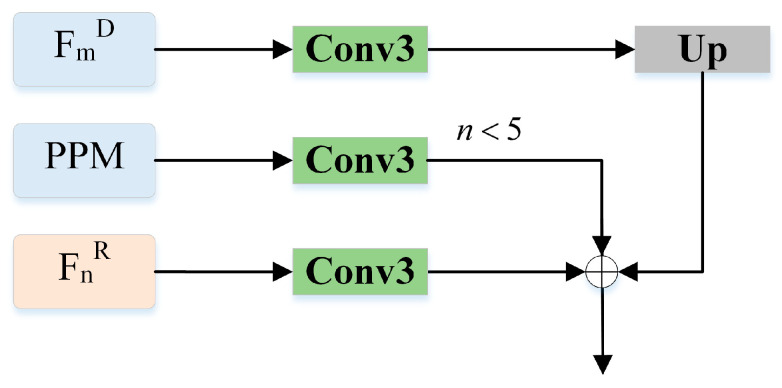
Fusion module with a direct summation.

Effectiveness of G^2^CMCSM. G^2^CMCSM plays an important role in the proposed G^2^CMCSNet. To verify the effectiveness of the G^2^CMCSM, we replaced the feature fusion module G^2^CMCSM with direct summation, as shown in Figure 4. We denote this evaluation as “A1” in Table 2. As can be seen in Table 2, G^2^CMCSM shows better overall performance than A1, which validates the effectiveness of G^2^CMCSM.

Effectiveness of DEM in G^2^CMCSM. To explore the effectiveness of the DEM, we remove it from our full model. We denote this evaluation as “A2” in Table 2. From the results in Table 2, it can be observed that the performance degrades without using DEM. This indicates the effectiveness of the DEM.

Effectiveness of REM in G^2^CMCSM. To explore the effectiveness of the DEM, we remove REM from our full model. We denote this evaluation as “A3” in Table 2. It can be observed that the performance degrades without using DEM. This indicates the effectiveness of the DEM.

Effectiveness of Global Guidance in G^2^CMCSM. To verify the effectiveness of the global guidance, we remove the PPM module from our full model. We denote this evaluation as “A4” in Table 2. As can be seen in Table 2, G^2^CMCSM shows better overall performance than CMCSM, which supports our claim that the global guidance role should be strengthened.

Effectiveness of Cross-Scale Strategy in G^2^CMCSM. To verify the effectiveness of the cross-scale strategy, we replace cross-scale fusion with corresponding level fusion, RGB features are enhanced by corresponding level depth map features rather than adjacent level depth map features. We denote this evaluation as “A5” in Table 2. G^2^CMCSM shows better overall performance than the model with corresponding level fusion, which validates the importance of cross-scale strategy.

## 5. Conclusions

In this paper, we have proposed a new G^2^CMCSNet for RGB-D SOD, which suppresses the distractors in the depth map and effectively fuses the features of RGB and depth maps. The G^2^CMCSNet consists of three main components: the DEM, which fuses the cross-modal cross-scale features extracted from the encoder; the REM, which enhances the details of SOD; and the GG, which suppresses the noise from unreliable raw depth. With all these modules working together, G^2^CMCSNet can effectively detect salient objects in complex scenarios. Extensive comparison experiments and ablation studies show that the proposed G^2^CMCSNet achieves superior performance on five widely used SOD benchmark datasets. In the future, we will extend the proposed models to other cross-modal tasks.

## Figures and Tables

**Table 1:** Quantitative comparison with SOTA models using MAE (M), S-measure ($S_{m}$), max F-measure ($F_{m}$), and max E-measure ($E_{m}$). ↑(↓) denotes that the higher (lower) is better. The best two results are highlighted in red and green.

	Metric	DMRA	CMW	PGAR	HAINet	JLDCF	DCF	DSA2F	DCMF	HINet	CFIDNet	SPSNet	C2DFNet	Ours
NLPR	*M*↓	0.031	0.03	0.025	0.024	0.022	0.024	0.024	0.029	0.026	0.026	0.024	0.021	0.021
	*S_m_*↑	0.899	0.917	0.93	0.924	0.925	0.922	0.918	0.922	0.923	0.922	0.923	0.928	0.928
	*F_m_*↑	0.888	0.912	0.925	0.922	0.916	0.917	0.917	0.913	0.915	0.914	0.918	0.926	0.927
	*E_m_*↑	0.941	0.94	0.953	0.956	0.962	0.954	0.951	0.939	0.948	0.95	0.956	0.957	0.960
SSD	*M*↓	0.058	0.052	-	0.052	0.053	0.054	0.047	0.054	0.049	0.051	-	0.047	0.043
	*S_m_*↑	0.857	0.875	-	0.857	0.861	0.852	0.876	0.882	0.865	0.879	-	0.871	0.880
	*F_m_*↑	0.858	0.883	-	0.859	0.862	0.858	0.878	0.88	0.874	0.882	-	0.883	0.892
	*E_m_*↑	0.898	0.909	-	0.895	0.889	0.892	0.911	0.905	0.903	0.916	-	0.912	0.917
STEREO	*M*↓	0.047	0.042	0.04	0.039	0.042	0.036	0.038	0.042	0.048	0.042	0.035	0.037	0.039
	*S_m_*↑	0.886	0.913	0.914	0.915	0.905	0.915	0.904	0.917	0.900	0.91	0.914	0.911	0.910
	*F_m_*↑	0.895	0.909	0.909	0.914	0.901	0.913	0.91	0.914	0.895	0.906	0.908	0.91	0.909
	*E_m_*↑	0.93	0.93	0.93	0.938	0.946	0.943	0.939	0.929	0.921	0.935	0.941	0.938	0.935
NJU2K	*M*↓	-	0.046	0.043	0.038	0.043	0.042	-	0.043	0.039	0.038	0.033	0.039	0.032
	*S_m_*↑	-	0.903	0.909	0.912	0.903	0.895	-	0.913	0.915	0.914	0.918	0.908	0.919
	*F_m_*↑	-	0.913	0.917	0.925	0.903	0.908	-	0.922	0.925	0.923	0.927	0.918	0.929
	*E_m_*↑	-	0.925	0.931	0.94	0.944	0.932	-	0.932	0.936	0.938	0.949	0.937	0.949
SIP	*M*↓	-	0.063	0.056	0.053	0.051	0.053	0.057	-	0.066S	0.051	0.044	0.052	0.044
	*S_m_*↑	-	0.867	0.876	0.879	0.879	0.872	0.861	-	0.856	0.881	0.892	0.871	0.890
	*F_m_*↑	-	0.889	0.892	0.906	0.885	0.899	0.891	-	0.880	0.9	0.91	0.895	0.912
	*E_m_*↑	-	0.9	0.904		0.923	0.915	0.909	-	0.887	0.918	0.931	0.913	0.927

**Table 2:** Ablation analysis on three datasets. The best results are highlighted in bold.

Models	RGBD135				SSD				SIP			
	*M*↓	*S_m_*↑	*F_m_*↑	*E_m_*↑	*M*↓	*S_m_*↑	*F_m_*↑	*E_m_*↑	*M*↓	*S_m_*↑	*F_m_*↑	*E_m_*↑
**Ours**	**0.020**	**0.920**	**0.931**	**0.959**	**0.043**	**0.880**	**0.892**	**0.916**	**0.044**	**0.890**	**0.912**	**0.927**
A1	0.025	0.907	0.925	0.939	0.048	0.860	0.859	0.90	0.051	0.877	0.900	0.915
A2	0.025	0.921	0.917	0.952	0.058	0.845	0.847	0.891	0.063	0.851	0.878	0.897
A3	0.024	0.905	0.919	0.936	0.047	0.868	0.881	0.902	0.045	0.888	0.912	0.926
A4	0.022	0.913	0.927	0.948	0.049	0.862	0.875	0.899	0.047	0.885	0.909	0.921
A5	0.021	0.915	0.929	0.951	0.053	0.865	0.868	0.898	0.046	0.885	0.913	0.922

## Data Availability

Not applicable.

## References

[1] Zhu J.Y., Wu J., Xu Y., Chang E., Tu Z. Unsupervised object class discovery via saliency-guided multiple class learning. Proceedings of the 2012 IEEE/CVF Conference on Computer Vision and Pattern Recognition (CVPR).

[2] Fan D.P., Wang W., Cheng M.M., Shen J. Shifting more attention to video salient object detection. Proceedings of the 2019 IEEE/CVF Conference on Computer Vision and Pattern Recognition (CVPR).

[3] Shimoda W., Yanai K. Distinct class-specific saliency maps for weakly supervised semantic segmentation. Proceedings of the European Conference on Computer Vision (ECCV).

[4] Mahadevan V., Vasconcelos N. Saliency-based discriminant tracking. Proceedings of the 2009 IEEE Conference on Computer Vision and Pattern Recognition.

[5] Li N., Ye J., Ji Y., Ling H., Yu J. Saliency detection on light field. Proceedings of the 2014 IEEE Conference on Computer Vision and Pattern Recognition.

[6] Lin T.Y., Dollár P., Girshick R., He K., Hariharan B., Belongie S. Feature pyramid networks for object detection. Proceedings of the 2017 IEEE Conference on Computer Vision and Pattern Recognition (CVPR).

[7] Wang X., Ma H., Chen X., You S. (2018). Edge preserving and multiscale contextual neural network for salient object detection. IEEE Trans. Image Process..

[8] Liu S., Huang D., Wang Y. Receptive field block net for accurate and fast object detection. Proceedings of the European Conference on Computer Vision (ECCV).

[9] Liu J.J., Hou Q., Cheng M.M., Feng J., Jiang J. A simple pooling-based design for real-time salient object detection. Proceedings of the 2019 IEEE/CVF Conference on Computer Vision and Pattern Recognition (CVPR).

[10] Zhao J.X., Cao Y., Fan D.P., Cheng M.M., Li X.Y., Zhang L. Contrast prior and fluid pyramid integration for RGBD salient object detection. Proceedings of the 2019 IEEE/CVF Conference on Computer Vision and Pattern Recognition (CVPR).

[11] Piao Y., Rong Z., Zhang M., Ren W., Lu H. A2dele: Adaptive and attentive depth distiller for efficient RGB-D salient object detection. Proceedings of the 2020 IEEE/CVF Conference on Computer Vision and Pattern Recognition (CVPR).

[12] Chen S., Fu Y. Progressively guided alternate refinement network for RGB-D salient object detection. Proceedings of the European Conference on Computer Vision (ECCV).

[13] Fan D.P., Zhai Y., Borji A., Yang J., Shao L. BBS-Net: RGB-D salient object detection with a bifurcated backbone strategy network. Proceedings of the European Conference on Computer Vision (ECCV).

[14] Li G.Y., Liu Z., Ye L.W., Wang Y., Ling H.B. Cross-modal weighting network for RGB-D salient object detection. Proceedings of the European Conference on Computer Vision (ECCV).

[15] Li G.Y., Liu Z., Chen M.Y., Bai Z., Lin W.S., Ling H.B. (2021). Hierarchical Alternate Interaction Network for RGB-D Salient Object Detection. IEEE Trans. Image Process..

[16] Zhou T., Fu H.Z., Chen G., Zhou Y., Fan D.P., Shao L. Specificity-preserving RGB-D Saliency Detection. Proceedings of the 2021 IEEE International Conference on Computer Vision (ICCV).

[17] Zhao H.S., Shi J.P., Qi X.J., Wang X.G., Jia J.Y. Pyramid scene parsing network. Proceedings of the 2017 IEEE Conference on Computer Vision and Pattern Recognition (CVPR).

[18] Wang L.Z., Wang L.J., Lu H.C., Zhang P.P., Ruan X. Saliency detection with recurrent fully convolutional networks. Proceedings of the European Conference on Computer Vision (ECCV).

[19] Qu L.Q., He S.F., Zhang J.W., Tian J.D., Tang Y.D., Yang Q.X. (2017). RGBD salient object detection via deep fusion. IEEE Trans. Image Process..

[20] Liu N., Han J.W. Dhsnet: Deep hierarchical saliency network for salient object detection. Proceedings of the 2016 IEEE Conference on Computer Vision and Pattern Recognition (CVPR).

[21] Chen S., Tan X., Wang B., Hu X. Reverse attention for salient object detection. Proceedings of the European Conference on Computer Vision (ECCV).

[22] Wang W., Shen J., Cheng M.M., Shao L. An iterative and cooperative top-down and bottom-up inference network for salient object detection. Proceedings of the IEEE/CVF Conference on Computer Vision and Pattern Recognition (CVPR).

[23] Wu Z., Su L., Huang Q. Cascaded partial decoder for fast and accurate salient object detection. Proceedings of the IEEE/CVF Conference on Computer Vision and Pattern Recognition (CVPR).

[24] Zhang L., Zhang J., Lin Z., Lu H., He Y. Capsal: Leveraging captioning to boost semantics for salient object detection. Proceedings of the IEEE/CVF Conference on Computer Vision and Pattern Recognition (CVPR).

[25] Chen H., Li Y.F., Su D. (2019). Multi-modal fusion network with multi-scale multi-path and cross-modal interactions for RGB-D salient object detection. Pattern Recognit..

[26] Zhang J., Fan D.P., Dai Y.C., Yu X., Zhong Y.Z., Barnes N., Shao L. RGB-D saliency detection via cascaded mutual information minimization. Proceedings of the 2021 IEEE International Conference on Computer Vision (ICCV).

[27] Ji W., Li J., Yu S., Zhang M., Piao Y., Yao S., Bi Q., Ma K., Zheng Y., Lu H. Calibrated rgb-d salient object detection. Proceedings of the 2021 IEEE/CVF Conference on Computer Vision and Pattern Recognition (CVPR).

[28] Lee M.Y., Park C.W., Cho S.W., Lee S.Y. SPSN: Superpixel prototype sampling network for RGB-D salient object detection. Proceedings of the ECCV.

[29] Sun P., Zhang W.H., Wang H.Y., Li S.Y., Li X. Deep RGB-D Saliency Detection with Depth-Sensitive Attention and Automatic Multi-Modal Fusion. Proceedings of the 2021 IEEE/CVF Conference on Computer Vision and Pattern Recognition (CVPR).

[30] Zhang C., Cong R., Lin Q., Ma L., Li F., Zhao Y., Kwong S. Cross-modality discrepant interaction network for RGB-D salient object detection. Proceedings of the 29th ACM International Conference on Multimedia.

[31] Wu Z., Gobichettipalayam S., Tamadazte B., Allibert G., Paudel D.P., Demonceaux C. Robust rgb-d fusion for saliency detection. Proceedings of the International Conference on 3D Vision (3DV).

[32] Qin X., Zhang Z., Huang C., Gao C., Dehghan M., Jagersand M. BASNet: Boundary-aware salient object detection. Proceedings of the 2019 IEEE/CVF Conference on Computer Vision and Pattern Recognition (CVPR).

[33] Fu K., Fan D.P., Ji G.P., Zhao Q. JL-DCF: Joint learning and densely-cooperative fusion framework for rgb-d salient object detection. Proceedings of the IEEE/CVF Conference on Computer Vision and Pattern Recognition (CVPR).

[34] Wang F., Pan J., Xu S., Tang J. (2022). Learning Discriminative Cross-modality Features for RGB-D Saliency Detection. IEEE Trans. Image Process..

[35] Bi H.B., Wu R.W., Liu Z.Q., Zhu H.H. (2023). Cross-modal Hierarchical Interaction Network for RGB-D Salient Object Detection. Pattern Recognit..

[36] Chen T.Y., Hu X.G., Xiao J., Zhang G.F., Wang S.J. (2022). CFIDNet: Cascaded Feature Interaction Decoder for RGB-D Salient Object Detection. Neural Comput. Appl..

[37] Zhang M., Yao S.Y., Hu B.Q., Piao Y.R., Ji W. (2022). C^2^DFNet: Criss-Cross Dynamic Filter Network for RGB-D Salient Object Detection. IEEE Trans. Multimed..

[38] Ju R., Ge L., Geng W.J., Ren T.W., Wu G.S. Depth saliency based on anisotropic center-surround difference. Proceedings of the IEEE International Conference on Image Processing (ICIP).

[39] Niu Y.Z., Geng Y.J., Li X.Q., Liu F. Leveraging stereopsis for saliency analysis. Proceedings of the 2012 IEEE Conference on Computer Vision and Pattern Recognition (CVPR).

[40] Peng H.W., Li B., Xiong W.H., Hu W.M., Ji R.R. RGBD salient object detection: A benchmark and algorithms. Proceedings of the European Conference on Computer Vision (ECCV).

[41] Zhu C.B., Li G. A three-pathway psychobiological framework of salient object detection using stereoscopic technology. Proceedings of the IEEE International Conference on Computer Vision Workshops (ICCVW).

[42] Fan D.P., Lin Z., Zhang Z., Zhu M., Cheng M.M. (2021). Rethinking RGB-D salient object detection: Models, data sets, and large-scale benchmarks. IEEE Trans. Neural Netw. Learn. Syst..

[43] Achanta R., Hemami S., Estrada F., Susstrunk S. Frequency-tuned salient region detection. Proceedings of the 2009 IEEE Conference on Computer Vision and Pattern Recognition (CVPR).

[44] Perazzi F., Krähenbühl P., Pritch Y., Hornung A. Saliency filters: Contrast based filtering for salient region detection. Proceedings of the 2012 IEEE Conference on Computer Vision and Pattern Recognition (CVPR).

[45] Fan D.P., Cheng M.M., Liu Y., Li T., Borji A. Structure-measure: A new way to evaluate foreground maps. Proceedings of the IEEE International Conference on Computer Vision (CVPR).

[46] Fan D.P., Gong C., Cao Y., Ren B., Cheng M.M., Borji A. Enhanced-alignment measure for binary foreground map evaluation. Proceedings of the International Joint Conference on Artificial Intelligence (IJCAI).

[47] Piao Y.R., Ji W., Li J.J., Zhang M., Lu H.C. Depth-induced multi-scale recurrent attention network for saliency detection. Proceedings of the IEEE International Conference on Computer Vision (ICCV).

